# Complete genome sequence of *Pseudomonas* sp. 20-5, an effective biocontrol bacterium isolated from soil

**DOI:** 10.1128/mra.00534-26

**Published:** 2026-06-29

**Authors:** Xinyue Li, Xiaoqing Li, Xiqian Lan, Junhua Hu, Jie Xie, Zeyang Zhou, Junhong Wei

**Affiliations:** 1 State Key Laboratory of Resource Insects, Southwest University 26463, Chongqing, China; 2 Chongqing Key Laboratory of Microsporidia Infection and Prevention, Southwest University 26463, Chongqing, China; 3 College of Sericulture, Textile and Biomass Sciences, Southwest University 26463, Chongqing, China; 4 National Citrus Engineering and Technology Research Center, Citrus Research Institute of Southwest University 117459, Chongqing, China; 5 College of Life Sciences, Chongqing Normal University 12524, Chongqing, China; DOE Joint Genome Institute, Berkeley, California, USA

**Keywords:** *Pseudomonas*, biocontrol, antifungal agents

## Abstract

Here, we report the complete genome sequence of *Pseudomonas* sp. 20-5, a potent biocontrol bacterium isolated from a soil sample of Chongqing, China. The genome consists of 6,335,883 bp with a GC content of 66.51%, in which 5,729 protein-coding genes, 12 rRNA, and 65 tRNA genes were identified.

## ANNOUNCEMENT

Plant diseases caused by bacteria and fungi pose a significant threat to agricultural production; the increasing use of chemicals to control plant diseases leads to deposits on crops and harvests, threatening both environmental health and food security (1). Biological control is an effective and sustainable alternative for plant disease control. Many studies have focused on the identification of biocontrol bacteria, with special emphasis on *Pseudomonas* (2, 3). In our previous work, *Pseudomonas* sp. strain 20-5 was isolated from a soil sample collected from Chongqing, China (29.812833 N, 106.414117 E) on 9 April 2000 (4). *Pseudomonas* sp. strain 20-5 exhibited strong biocontrol ability against plant pathogens, it can suppress mycelium growth of *Phytophthora infestans*, protect potato seedlings and tomatoes from *P*. in*festans* infection, or reduce its severity in field experiments (5). Genome sequence information is essential for understanding the antagonistic mechanism of strain 20-5.

To isolate the microbes, the soil sample was resuspended in 1 mL sterilized normal saline followed by 10-fold serial dilution to 10^−6^. Then, 100 μL of each dilution was added to LB () liquid medium (6) and incubated at 28°C for 1 day. To achieve an axenic culture, three iterations of subculturing were conducted from a single colony. The single colony of *Pseudomonas* sp. strain 20-5 was isolated and stored in 15% glycerol at –80°C until use.

Strain 20-5 was inoculated in 5 mL LB liquid medium for 16 h at 30°C to provide cell biomass for DNA extraction. Total DNA was extracted using the bacterial DNA extraction kit T07-100 (Majorbio, Shanghai, China). Genomic DNA was fragmented to 10 kb with G-tube (Covaris, USA), size distribution was verified with Agilent Technologies 2100 Bioanalyzer. Then, the total DNA was subjected to whole-genome sequencing at Majorbio Technologies Co. Ltd. (Beijing, China). SMRTbell prep kit 3.0 (Pacific Biosciences, CA) was used to for library preparation. PacBio sequel IIe system was used to sequence the genome following manufacturer’s recommendations (Pacific Biosciences, CA). PacBio CCS sequencing mode generated 50,353 raw reads with a read N_50_ 11,312. Fastp v0.20.0 was used for quality control, the filtered subreads were assembled by Unicycler v0.4.8, and the resulting contigs were polished by Pilon v1.22 (7–9). The genome assembly was assessed for completeness using BUSCO v5.8.0 (10). Protein-, rRNA-, and tRNA-coding genes were annotated using the NCBI Prokaryotic Genome Annotation Pipeline v6.7 (11). To determine the taxonomic position of strain 20-5, genome comparison was performed using the Type Strain Genome Server (TYGS) (12). Analysis of biosynthetic gene clusters (BGCs) was performed using antiSMASH bacterial version 8.0 (13). Default parameters were used except where otherwise noted.

A complete circular genome was assembled, with a length of 6,335,883 bp and a G + C content of 66.51%, the average genome coverage was 89.67×. BUSCO assessment of the final assembly showed 99.0% completeness (C: 99.0% [S: 99.0%, D: 0.0%], F: 0.0%, M: 1.0%). The genome annotation identified 5,898 genes, consisting of 5,729 coding sequences (CDS), 65 tRNAs, 12 rRNAs, and 92 sRNAs. The analysis showed that strain 20-5 belongs to *Pseudomonas aeruginosa*, the digital DNA-DNA hybridization (dDDH) values between the query genome and the type strain *Pseudomonas aeruginosa* DSM 50071 were 97.9% (d₀), 95.5% (d₄), and 98.6% (d₆). antiSMASH identified 14 BGCs, comprising 5 NRPS clusters, 1 PKS cluster, and 8 other clusters. Strain 20-5 possesses the biosynthetic clusters of pyoluteorin and pyochelin, which may account for its antagonistic activities. The complete genome sequence of strain 20-5 will accelerate further study on the antifungal mechanism of this strain.

## Data Availability

The genome sequence of *Pseudomonas* sp. 20-5 has been deposited in GenBank under accession number CM165584.1. The sequencing read data sets and the assembly of Pseudomonas sp. 20-5 have been deposited in the NCBI database under Bioproject PRJNA1450866 and Biosample SAMN57173361. The SRA accession number used for assembly is SRR38897649.
